# Hospital meals are existential asylums to hospitalized people with a neurological disease: A phenomenological–hermeneutical explorative study of the meaningfulness of mealtimes

**DOI:** 10.1002/nop2.246

**Published:** 2019-02-21

**Authors:** Malene Beck, Regner Birkelund, Ingrid Poulsen, Bente Martinsen

**Affiliations:** ^1^ Institute of Health, Department of Nursing Science Aarhus University Copenhagen Denmark; ^2^ Neurology Department Zealand University Copenhagen Denmark; ^3^ Section of Health Services Research Lillebaelt Hospital University of Southern Denmark Vejle Denmark; ^4^ Vejle Hospital Vejle Denmark; ^5^ Institute of Public Health, Department of Nursing Science Aarhus University Aarhus Denmark; ^6^ TBI Unit, Research Unit on Brain Injury Rehabilitation, RUBRIC, Department of Neurorehabilitation Glostrup Hospital, Copenhagen University Hvidovre Denmark

**Keywords:** aesthetics, appetite, care, disease, environment, hermeneutics, hospital, mealtimes, neurology, nursing, patients, phenomenology

## Abstract

**Aim:**

Hospital meals are challenging for neurological patients. Patients struggle with both physical eating disabilities and social issues during mealtimes. The aim of this study was to examine the meaningfulness of the phenomenon of hospital meals for hospitalized patients with a neurological disease.

**Design:**

Interviews (*N* = 23) with neurological patients were analysed and interpreted to gain in‐depth comprehensive knowledge of the phenomenon of hospital mealtimes.

**Method:**

Data were analysed and interpreted in a three‐phased process using a phenomenological–hermeneutic approach inspired by Paul Ricouer.

**Results:**

Four themes were identified: (a) A lonely ride together with others; (b) Letting the chaotic setting fade into the background; (c) Mechanical activity with great personal significance; and (d) Humanizing the setting when eating in the hospital. Mealtimes were supporting existential moments to patients. Offering a calm mealtime setting was experienced by the patients as an asylum where uplifting and comforting feelings were raised.

## 1 INTRODUCTION

“To patients, hospitalization often is a comma in their life.” This quotation is from an informal meeting between health professionals at a Danish hospital discussing the importance of mealtimes to hospitalized patients with a neurological disease. However, for neurological patients, hospitalization may be considered “a full sentence in their life” allowing them time to breathe (in a figurative way), before continuing with their lives (Beck, Birkelund, Poulsen, & Martinsen, 2017; Beck, Martinsen, Poulsen, & Birkelund, 2016; Beck, Poulsen, Martinsen, & Birkelund, 2017). This study is an elaboration of two previous studies (Beck, Birkelund et al., 2017; Beck, Poulsen et al., 2017). The aim of this study was to show how the phenomenon of mealtimes is meaningful to hospitalized patients with a neurological disease.

## 2 BACKGROUND

The impact of getting a neurological diagnosis is extensive and affects humans on different levels. When patients with neurological diseases are hospitalized, they are all confronted with various impairments and disabilities that influence their nutritional intake, possibly leading to risk of malnutrition (Hafsteinsdóttir, Mosselman, Schoneveld, Riedstra, & Kruitwagen, 2010; Westergren, Ohlsson, & Hallberg, 2001). Eating disabilities are characterized as either concrete physical disabilities, such as dysphagia or paralysis, or neuropsychological impairments, such as apraxia or trouble handling the food on the plate (Medin, Windahl, Arbin, Tham, & Wredling, 2011).

Patients with neurological diseases constitute one of the largest groups experiencing eating disabilities. Kumlien & Axelsson (2002) showed that patients who have had a stroke have an especially high number of eating disabilities; more than 80% of these patients in nursing homes were assessed to have some kind of dependence when eating. Many inpatients with neurological diseases experience more than one eating disability (Westergren et al., 2001). These eating disabilities often cause a lack of participation in meals with relatives or in meals with other/fellow patients. However, for patients admitted to hospital, it can be difficult to decide whether mealtimes should be private or whether the meals should be shared with other fellow patients (Sidenvall, Fjellstrom, & Ek, 1996, Manthorpe & Watson 2003). Hence, conventions, habits and involvement of other people tend to form the basis of mastering eating situations in patients hospitalized with a neurological disease (Martinsen & Norlyk, 2012; Medin et al., 2011). Having conversations and socializing during mealtimes address some of the main issues regarding malnutrition among patients suffering from a neurological disease.

There is a growing interest in the patients’ experiences of mealtimes in hospitals to improve the existing hospital mealtime situation. Several studies have explored hospital mealtime experiences using various methods of collecting empirical data (Holst, Mortensen, Jacobsen, & Rasmussen, 2010; Lassen, Kruse, & Bjerrum, 2005; Naithani, Whelan, Thomas, Gulliford, & Morgan, 2008; Ottrey, Porter, Huggins, & Palermo, 2018). However, research on the neurological context of patients’ experiences of hospital mealtimes is sparse. Moreover, existing research mainly focuses on patients’ experiences with eating difficulties, whereas the practical element of the mealtime situation and its relational and aesthetic importance have not received similar attention. Life with eating difficulties is a complex phenomenon where the social dimension of mealtimes is particularly challenged; hence, patients suffering from neurological diseases experienced mealtimes as a disgusting, uncomfortable long‐term experience that depended on help from others and causing them to feel embarrassment (Medin et al., 2011, Carlsson, Ehrenberg, & Ehnfors, 2004). In addition, altered physical and social appearance is related to difficulties preparing and transporting food to the mouth, as are swallowing deficits (Perry, & McLaren, 2004).

Moreover, studies have demonstrated that the long‐term mealtime experiences of patients suffering from a neurological disease involve existential issues because the eating disability causes them feelings of being abandoned and struggling with loss, which make them strive towards a normal life (Perry, Hamilton, Williams, & Jones, 2013, Carlsson, Ehrenberg, & Ehnfors, 2004). However, in‐depth qualitative knowledge of neurological patients’ hospital mealtime experiences is limited. Hence, more knowledge is needed of how to bridge the gap between what is known about malnutrition in complex healthcare organizations and how this knowledge can be used to create meaningful, practical and sustainable changes to patients’ mealtime experiences.

## 3 PURPOSE

To explore how the phenomenon of mealtimes is meaningful to hospitalized patients with a neurological disease.

## 4 METHOD

This study is a qualitative study that gives in‐depth and comprehensive analysis of the phenomenon of hospital mealtimes based on data from earlier studies (Beck, Birkelund et al., 2017; Beck et al., 2016; Beck, Poulsen et al., 2017). This study has a phenomenological–hermeneutic approach inspired by the French philosopher Ricouer (1973, 1979, 1984). According to Ricouer, analysis and interpretation can be considered as an endless hermeneutic spiral, providing new insight into a phenomenon (1984). Thus, this study offers an in‐depth analysis and contributes to a comprehensive understanding of the phenomenon of hospital mealtimes and thus adds to the existing findings focused on patients’ experiences of hospital meals. The study complied with the Consolidated Criteria for Reporting Qualitative Research (COREQ) (Tong, Sainsbury, & Craig, 2007).

### 4.1 Participants

A total of 23 inpatients with a neurological disease participated in interviews regarding their experiences of hospital mealtimes (Beck, Birkelund et al., 2017; Beck, Poulsen et al., 2017). The participants were introduced to the aim of the study when they were approached face‐to‐face by the first author (MB). All of the invited patients accepted to participate. The number of participants was considered to give insight and a “richly textured understanding” of how patients assign meaning to the mealtime activity before and after an intervention changing mealtime settings (Kvale & Brinkmann, 2009). The participants had different neurological diagnoses including migraine, Guillain–Barre syndrome, stroke and multiple sclerosis. Some patients were totally dependent on caregivers, while others varied between needing full assistance during meals and being able to eat by themselves. The participants varied in age, diagnoses, days of admission and eating disabilities. The participants were selected based on an assumption of being most likely to contribute to the conversation with information‐rich data. Patients with severe cognitive deficits, expressive aphasia and dementia were excluded. Further characteristics of participants are illustrated in Table 1.

**Table 1:** Overview of the characteristics of the participants

ID	Age & Gender	Symptoms and signsh	Dependent on assistance	Days of admission
1	76—Woman	Nausea Vomiting	1 person	>14
2	67—Woman	Unilateral paralysis	No	1
3	72—Man	Loss of appetite	No	3
4	78—Man	Paresis and decreased functions in right arm.	1 person	>14
5	30—Man	Paralysed No functions in arms or legs	2 persons	10
6	78—Man	Weakened functions in arms and hands	1 person	7
7	41—Man	None	No	3
8	54—Woman	Shaking and weakened arm functions	No	2
9	27—Woman	Loss of appetite	No	1
10	62—Woman	None	No	2
11	45—Woman	Scleroses	No	5
12	55—Woman	Tumour	1 person	3 Readmitted
13	65—Woman	Apoplexies	1 person	7
14	55—Man	Apoplexies	1 person	6
15	31—Woman	PNES (psychogenic nonepileptic seizures)	1 person	7
16	50—Woman	Tumour	No	>21
17	80—Man	Apoplexies	No	2
18	70—Man	Dizziness	1 person	>14
19	83—Woman	Apoplexies	No	3
20	80—Woman	Apoplexies	1 person	4
21	30—Woman	Epilepsy	1 person	2 Readmitted
22	38—Woman	Migraine	No	3
23	83—Man	Apoplexies	No	3

### 4.2 Data collection

According to Ricoeur, the possibility of analysis and interpretation is endless (Ricouer, 1979). This paper offers an in‐depth analysis and comprehensive understanding of the primary data investigating the phenomenon of mealtimes in a Danish neurological department in 2015. The data material consisted of large amounts of transcriptions, and the previous analysis reported in Beck, Birkelund et al. (2017) a&b) was far from exhaustive. Therefore, the authors returned to the material to get new insights. To achieve a nuanced understanding of patients’ experiences of meals in a neurological department, participants were interviewed individually (Brinkmann, 2013; Kvale & Brinkmann, 2009). The interviewer (MB) is experienced in interviewing; however, a semi‐structured interview guide was used to ensure that the relevant questions related to the phenomenon were posed (Kvale & Brinkmann, 2009). The interviewer memorized the interview guide and strived to make the interview seem like a conversation. The interviews started with an open question such as, *“Tell me a little about yourself. What is your story in relation to this place (the hospital)?” *Attention and responsiveness to the patients were expressed by asking relevant follow‐up questions to allow the patients to elaborate on all of the dimensions to their stories (Fog, 2007). The interviews were conducted at the hospital department in a suitable room and lasted between 45–60 min. The interviews were recorded, listened to and transcribed afterwards.

### 4.3 Ethical considerations

Written and verbal information about the study was given to all participants, and informed consent was obtained. Participants were informed that their names and other personal information would be anonymized to maintain confidentiality. They were reminded that they could withdraw from the study at any time without any consequences for their treatment and care in the department. This study was approved by the Danish Data Protection Agency and performed in accordance with the ethical guidelines of the Nordic Nurses Federation and the Helsinki Declaration.

### 4.4 Analysis and interpretation

Data analysis and interpretation were inspired by Ricoeur (,^1973^, ^1979^), and the aim was to obtain new knowledge about the phenomenon of hospital meals based on patient's experiences when hospitalized with a neurological disease. Three methodological phases were used during the analysis and interpretation. The data are presented more systematically in the paper than it was actually performed, due to the dialectical movement (Ricouer, 1979).

The analysis was based on a naïve reading (phase 1), where the text was read and reread to gain an intuitive understanding of the material. Through the structural analysis (phase 2), the text was structured into meaningful units. Thus, it became possible to visibly identify what the text was about and central themes. A rich description of the meaning units was considered crucial as they included important arguments for validating the impression derived from the naïve reading and a credible way to start interpretation of data. During the comprehensive understanding and discussion (phase 3), theoretical perspectives and relevant empirical research were included to give new insights and interpretation of the data.

## 5 RESULTS

### 5.1 Naïve reading

The naïve reading phase provided the overall impression that hospitalization increased the significance of mealtimes to patients. Being hospitalized with a neurological disease was an existential experience in patients’ everyday lives that they found difficult to embrace. Mealtimes represented a familiar and recognizable activity. The naïve reading indicated that comfort and well‐being were embedded in the mealtime activity. Comfort and well‐being were, however, dependent on how the way healthcare professionals managed the high‐paced environment and workload during mealtimes.

### 5.2 Structural analysis

Four themes were identified: (a) A lonely ride together with others; (b) Letting the chaotic setting fade into the background; (c) Mechanical activity with great personal significance; and (d) Humanizing the setting when eating in the hospital.

### 5.3 A lonely ride together with others

Patients described how the neurological disease was a life‐changing event and followed by many negative thoughts and feelings. Some of the aspects of living a life with a neurological disease were described like this:

To me, the disease meant that I had a swelling in the brain, which meant that I could not speak or remember some words. I have been sad, because I am afraid. You get a bit frightened and you are snapped out of it. This means that you do not want to see anyone because they should not see how I have looked (P18).

Existential issues in particular were embedded in the mealtime activity because eating in the hospital was described as a lonely ride. The patients were confronted with their life being different after being diagnosed with a neurological disease. Fore example when afflicted by a stroke the patients was confronted with a life with eating disabilities inwhich was futher exemplified during the mealtime activity. In that sense, hospital meals represented a concrete activity to the patients that reminded them of their life before the neurological diagnosis, but it was also a new challenge that the patients had to cope with because of their diagnoses. Some of the emotions at stake when eating in the hospital were illuminated when patients were confronted with eating disabilities, such as trouble handling a fork. The patients described how they could be mad, frustrated or sad during mealtimes. Hunger, appetite and satisfaction were identified as sensations that patients found difficult to relate to during hospitalization and contributed to making the mealtime situations problematic.

The patients explained how mealtimes often felt rather lonely. The loneliness, however, was considered a private matter, and patients did not share their thoughts of loneliness with relatives. Furthermore, the patients did not want to bother or burden their relatives by asking them to come visit them during mealtimes. The patients explained how they preferred their relatives to stay at home so they would not see how they struggled during mealtimes in the hospital. Persuading relatives to stay away meant that it was important to establish other situations for human contact enabling the sharing of thoughts or just being together. Here, the mealtime was an obvious possibility, representing a way to enhance the diversity of the patient experience and to experience togetherness. A woman gives an example:

I usually get out and pick up some food for Gitte (another patient). Normally she gets her food when I have finished. Yesterday I asked if we could eat together. It was nice. She does not speak (due to aphasia?), but at least we were together (P13).

The patients found a kind of community with their fellow patients established by identifying each other's loneliness during the mealtime situations. The community was not necessarily formed by those in the same room but could be formed together with other patients in the department. Being able to find a community with others during mealtimes was significant to the patients so they could feel “normal” and be in contact with life as it is outside the hospital. Hence, patients referred to how they normally would share meals with others, such as their colleagues at work, to achieve a positive experience during the day. However, establishing a community depended on the way healthcare staff managed the mealtime situations.

Nurses played an essential role in creating a positive basis for the patients’ mealtime experience. Making the mealtimes reflect the situation patients knew from outside the hospital made the mealtime situation in the hospital more recognizable to the patients. Thus, the commitment and compassion of staff when serving meals were crucial to make the patients’ mealtime experience positive. A woman describes the importance of the mealtimes where staff took the time to prepare and serve the meal with respect to her wishes:

When they (the staff members) take their time, you feel that they are not just at work. That they actually care for you and care about who I am. It might be that they don't care in reality, but you feel much less like a patient that way (P19).

### 5.4 Letting the chaotic setting fade into the background

The mealtime setting was described as a fast‐paced environment; some patients compared the eating environment to a railway station. Patients also elaborated on how mealtimes could be experienced as dedicated activities with only one purpose: nurturing. However, patients explained how they often experienced being disturbed while eating, which is a complete contrast to the main aim of the meal.

The uncertainty of not knowing when they would be disturbed created a feeling of being unsafe and made the patients anxious because they never had a private moment. A woman gives an example: *“I never know if I am disturbed in here. There is no such thing as a real break. You are always like, “are they coming to say something important now?” You do not really get rid of that feeling” (P19).* The patients in hospital described how they were put on hold and were always waiting for somebody to come and give them information. Being on hold meant that relaxation was suspended. Mealtimes decreased the feeling of being on hold because during this particular activity, the patients were physically seated and the only purpose was to wait for the food to be served.

Patients experienced that nurses did not manage to establish a relaxing and community‐creating mealtime setting. In these situations, the mealtime experience was in contrast to the experience of the mealtime activity outside the hospital, where patients would be used to relax. Therefore, when staff members managed to establish a setting with peace and quietness, the patients experienced relief related to the mealtime activity. Peace and quiet was described as a key element to calm the patients down, not only during mealtimes but also during the day. A woman described how the calmness during mealtimes influenced the quality of the rest of her day by saying:

Peace and quietness are to me related to a break of the day. Calmness is nice when I sit here and eat. I can relax better when they do not constantly walk back and forth. Well, just relax in my mind. I feel much better if I get to relax a bit during the day instead of driving through it in a high gear… up here (point to the ceiling) all day. Then… then it can be difficult to sleep at night (P15).

The patients’ experiences of the chaotic mealtime setting were obscured across time. The material showed that patients developed strategies to minimize the negative sensory impressions and to maintain a positive focus during the activity. For example, the patients described how they had learned to find ways to find happiness during mealtimes if calmness was not established. The patients explained that in the absence of other dining facilities, they preferred to eat in their bed, even though this practice was often contrary to staff recommendations.

For example, a patient reported that when she was asked to leave her bed in the morning to eat breakfast at a table in the hallway, she answered by saying: “*No thanks, I prefer to stay in my bed. That is where my place is. In my bed, I feel more comfortable. This is also where (in the bed) I eat my breakfast, when I am at home (P9).” *The bed was identified as a place where well‐being was the focus and the chaotic settings faded into the background. To the patients, the bed was a place where they were able to decide their own pace when eating and at the same time avoid human contact, such as eye contact, while eating. A woman gives this example:

When you are rushed, you may feel that… now I have to finish, because now they (the staff) have to finish and clean up. That is a big invasion of your privacy. I know I'm in an institution, but still… it is just nice to do things at my own pace (P19).

During the interviews, it was noted that peace and quiet was difficult to achieve during mealtimes. However, when peace and quiet was achieved, it revitalized the patients’ actions related to the meal. For example, the patients made their bed and cleaned up their room and—in this way—prepared themselves for the meal.

### 5.5 Mechanical activity with great personal significance

Well‐being was based on many sensory impressions experienced in the hospital by patients. Therefore, the impressions from the mealtime environment and especially the interaction with the staff (their body language, their tone of voice or their choice of words when serving meals) were crucial in relation to the patients’ ability to experience mealtimes as safe and pleasant. Thus, serving a meal to a patient without asking about the patients’ preferences or making eye contact meant that the patients experienced the mealtimes as being rather mechanical and without any aspects of well‐being. For example, the patients described this scenario as if the staff were at an industrial assembly line when serving their meals.

The fact that the mealtime was handled at the same time as other tasks such as drug rounds, doctor's rounds, answering phone calls and helping other patients to the toilet meant that the patients had to compete for the attention of the staff during mealtimes. These competing demands could be experienced as if the mealtimes had no priority at all. One of the patients said:

Mealtimes are not important in here. To the staff members, the mealtimes are just “muzak” (P9). The food is good enough here… but we cannot be proud of how we treat people in here (pointing at nurses); they have so much to do that they don't manage half of it (P1).

When the mealtime activity provided a calm and appealing environment, the patients related the mealtime to an experience of well‐being. The acknowledgement of calmness being related to well‐being during mealtimes made the patients determined to establish peace and quiet to make the activity more enjoyable. For example, if the patients were comfortable with their fellow patients, they valued sharing a meal together with them.

Patients described how just sitting together and eating, without having long conversations, could be a peaceful activity. In that sense, mealtimes were identified as activities that could personalize the hospitalization setting by connecting the hospital stay to positive (mealtime) experiences with others. This was not connected to the food itself but was related to the possibility of doing small talk with other people that did not involve some of the serious thoughts they had about their situations and their lives. Small talk could be seen as superficial but was important in the everyday life of the patients. Hence, it made time pass quickly and connected the patients to life outside the hospital because they often talked about former jobs and careers, children or other personal issues. Patients could talk about food preferences and mealtime experiences and talk about their life prior to their diagnosis. A woman describes how a simple menu could create a kind of community among strangers:

This month, there are menus, flowers and napkins on the tables. It makes it cozier. It creates an atmosphere and makes it more positive. People start talking and imagining what they want for dinner. You can already see when the people enter the hallway… then... they just have seen the menu before they do anything else (P12).

### 5.6 Humanizing the setting when eating in the hospital

In addition to the importance of having a relationship with fellow patients, conversations with staff during mealtimes were perceived as key activity to generate a positive experience. The conversations could be rather simple and had a common polite nature, such as talking about how many kids the staff members had or what they would do after their shift was over. However, despite the superficial nature of these conversations, they became important elements to make the mealtime activity a pleasant experience for patients because they acknowledged the patients’ need to get to know the staff. Getting to know caregivers as people and not just as professionals was comforting to patients because it influenced their feelings of safety during their hospitalization.

Furthermore, it was identified that the information that staff shared with their patients helped to make the relation between patients and staff equal, as the “white coat” serving the meal to the patients became personalized. The mealtime was an activity where the relationship between the patients and staff members could be strengthened (or weakened) depending on the interaction during mealtimes. A man elaborates:

I have been here for a long time and I know the first name of most of them (the nurses). Well, it is nice just to feel like you are a part of them. Then, they come by, say “hello” and so on and you get to know people across the shifts (P12).

Getting closer to staff through the meal activities meant that patients felt more like humans in their “role” as patients. In this way, the purpose of the meal was changed. The activity was no longer about nutrition but rather about human nourishment because the mealtime became an existential experience of life with a neurological disease. The mealtime was identified as a way to legitimize the patients’ needs to be vulnerable because the patients, especially during the mealtime activity, were more receptive to the support and care they received while eating, establishing an uplifting and comforting experience. A patient illustrates this feeling by saying:

The meal to me can be very relaxed. Mealtimes is like a moment in everything—even for those sitting and feeding you. They can also sit, relax, talk a little and get to know the patients. It is such a good way to get in touch and create a relationship. You also need to have that relationship with them (the nurses?). Now I know all the names of the nurses and their children. When they just sit and have a chat with you, it becomes a matter of not only being fed, but also a matter of having a positive experience (P5).

### 5.7 Comprehensive understanding and discussion

Based on the findings of this study, it can be argued that mealtimes form the basis for creating a community among humans and deinstitutionalize the mealtime activity. Studies exploring in the meaning of the phenomenon of mealtimes in people's everyday lives identify mealtimes as important social events with family and friends and as an essential part of human social life (Holm & Kristensen, 2012). From the beginning of life, food has been associated with relationships with others. Thus, eating habits are recognized as a part of our identity and role in society (Holm & Kristensen, 2012). Our study illustrated how mealtimes had the potential to make the relationships between strangers more intimate. Although mealtimes were shared with other patients and not relatives, mealtimes created a community among patients, which countered the patients’ feelings of mealtimes as being a “lonely ride.”

Our study showed that patients experienced a feeling of togetherness with others through the mealtime activity, which can be explained by the pause that the mealtime activity represented. Winther (2012) argues that pauses or breaks during the day give humans with an oasis where intimacy and a more personalized relationship with strangers are possible. Based on the studies of Kofod (2008) and Martinsen and Norlyk (2012), mealtimes in institutions can be considered a social activity. Our study supports these findings and gives new knowledge by illustrating the aesthetic dimension related to socialization during mealtimes in institutions. It was shown that a calm and appealing environment could give dignity to humans when eating together with strangers in the hospital. Hence, environments shared with well‐known fellow patients had the potential to personalize the mealtime activity and create a new kind of deinstitutionalized relationship with the staff (Ottrey et al., 2018).

Thus, our study underlines the importance of the sensory impressions of the mealtime environment. Sensory impressions from the human participants in the mealtime environment, especially related to the nurses’ behaviour, tone of voice and choice of words, played a crucial role in relation to the patients’ mealtime experience. Hence, a calm and appealing mealtime setting where nurses humanized the mealtime activity with socialization and aesthetic elements influenced the patients’ perceptions of the meal, thus converting the activity to a positive activity shared with others in a safe and comfortable way. Our study gives new insights into the patients’ existential needs for an aesthetic environment to mobilize recognizable patterns throughout the meal. This proved to be an unobtrusive and welcoming experience with patients being an active and integrated part of the meal activity.

Important existential dimensions influenced the patient's reassembled life in the hospital. Thus, patients strove for activities that were comparable to the lives they knew from outside the hospital. Mealtimes represented a familiar and recognizable activity that could be the highlight of their day and were described as existential sanctuaries during hospitalizations. Thus, based on the findings in this study it can be argued that if staff made mealtimes calmer without interruptions, mealtimes would give opportunities where the patients could allow their thoughts to wander off. No other daily activities in the hospital offered the patients the same opportunity.

Our findings illuminate the importance of mealtimes in relation to patients’ opportunities to experience well‐being. In our study, well‐being was related to the mealtimes as they made it possible for the patients to dwell. To the patients, mealtimes became an activity where objective time did not matter. Todres and Galvin (2010) highlighted that humans experience time as it flows. Our study showed that the patients often felt like they were in a hurry. This is in line with the ethnographic exploration of the mealtime environment by Ottrey et al. (2018), where the mealtime environment is characterized as a busy, disharmonious environment in relation to the strategy of patient‐centred (mealtime) care. Being in a hurry is linked to objective time. However, if calmness was created during mealtimes, it provided a certain atmosphere where the patients’ perceptions of well‐being stepped into the foreground and made the chaotic hospital setting fade into the background.

This knowledge gives new insights into the phenomenon of mealtime‐related nursing. Based on our findings, facilitating an environment where patients could “just be” for a while and let their thoughts wander off can be considered a necessity during illness. Thus, supporting patients in establishing a calm mealtime setting changes the purpose of the hospital meal. Mealtimes then become asylums where patients decide their own pace, sphere and thoughts. Thus, sensing calmness while eating in the hospital creates a space where contemplation can be conducted privately while being nourished.

### 5.8 Study limitations

We collected data in only one department at one hospital. Nevertheless, it can be argued that our findings have some generalizability and transferability and may be relevant to other departments with patients suffering from comparable medical diseases. Generalizability should be seen in relation to the typical features of the phenomenon that has been described by this phenomenological–hermeneutic study (Polit & Beck, 2010). This study may be strengthened by including healthcare staffs’ perspectives on the mealtime phenomenon to shed light on the importance of the professionals’ mealtime actions for hospitalized patients. Including patients with dysphagia, nasogastric feeding and PEG feeding may also strengthen the study since these groups of patients have severe eating disabilities. Their perspectives could unfold the phenomenon of mealtimes in new ways, hence adding further variation in relation to the recruitment of participants (Malterud, 2011).

## 6 CONCLUSION

The phenomenon of mealtimes was significant to hospitalized patients with a neurological disease because mealtimes supported patients existentially during hospitalization with a neurological disease. A peaceful eating hospital environment was identified as crucial. Offering a calm mealtime setting allowed the patients to experience an asylum where supporting, uplifting and comforting feelings were raised. Patients were able to be attuned to their surroundings and to be in touch with their senses, which they considered meaningful and promoting well‐being. Patients’ mealtime experiences, however, depended on how the staff members orchestrated the activity and whether they included both a human and an aesthetic approach when serving meals to the patients. This meant that patients strived for a more personal and equal relationship with the healthcare professionals to ensure a positive experience during their hospitalization.

## 7 RELEVANCE TO CLINICAL PRACTICE

This study highlights that patients are existentially challenged during mealtimes in hospitals. Patients suffering from a neurological disease assign great importance to their surroundings during mealtimes and experience calm and appealing environments as an existential way to support life with a neurological disease. When building new hospitals or renovating existing, it is important to consider patient‐centred mealtime areas as environments that make existential care possible. Additionally, healthcare professionals need to consider that a mealtime means much more than food on the plate to patients and that it in fact is an important aspect of their care.

## ETHICAL APPROVAL

The study was approved by the Danish Data Supervisory Committee by Region Zeeland (REG‐16‐2013).

## CONFLICT OF INTEREST

None declared.

## AUTHOR CONTRIBUTIONS

M.B, R.B, I.P and B.M: Study design, analysis and manuscript preparation. M.B: Data collection.
